# Bleeding risk and mortality according to antithrombotic agents’ exposure in cancer-related stroke patients: nationwide population-based cohort study in South Korea

**DOI:** 10.1186/s12883-023-03208-4

**Published:** 2023-05-09

**Authors:** Bo Kyu Choi, Ji Sung Lee, Hae Reong Kim, Han Sang Kim, Yo Han Jung, Yu Rang Park

**Affiliations:** 1 Department of Biomedical Systems Informatics, Yonsei University College of Medicine, Seoul, Republic of Korea; 2 Clinical Research Center, Asan Medical Center, Asan Institute for Life Sciences, University of Ulsan College of Medicine, Seoul, 05505 Korea; 3 Yonsei Cancer Center, Division of Medical Oncology, Department of Internal Medicine, Yonsei University College of Medicine, Seoul, Korea; 4 Graduate School of Medical Science, Severance Biomedical Science Institute, Brain Korea 21 Project, Yonsei University College of Medicine, Seoul, Korea; 5 Department of Neurology, Gangnam Severance Hospital, Yonsei University College of Medicine, Seoul, Korea

**Keywords:** Cancer, Stroke, Antithrombotics

## Abstract

**Background:**

Ischemic stroke with active cancer is thought to have a unique mechanism compared to conventional stroke etiologies. There is no gold standard guideline for secondary prevention in patients with cancer-related stroke, hence, adequate type of antithrombotic agent for treatment is controversial.

**Methods:**

Subjects who were enrolled in National Health Insurance System Customized Research data during the period between 2010 and 2015 were observed until 2019. Subject diagnosed with ischemic stroke within six months before and 12 months after a cancer diagnosis was defined as cancer-related stroke patient. To solve immeasurable time bias, the drug exposure evaluation was divided into daily units, and each person-day was classified as four groups: antiplatelet, anticoagulant, both types, and unexposed to antithrombotic drugs. To investigate bleeding risk and mortality, Cox proportional hazards regression model with time-dependent covariates were used.

**Results:**

Two thousand two hundred eighty-five subjects with cancer-related stroke were followed and analyzed. A group with anticoagulation showed high estimated hazard ratios (HRs) of all bleeding events compared to a group with antiplatelet (major bleeding HR, 1.35; 95% confidence interval [CI], 1.20–1.52; *p* < 0.001). And the result was also similar in the combination group (major bleeding HR, 1.54; 95% CI, 1.13–2.09; *p* = 0.006). The combination group also showed increased mortality HR compared to antiplatelet group (HR, 1.72; 95% CI, 1.47–2.00; *p* < 0.001).

**Conclusions:**

Bleeding risk increased in the anticoagulant-exposed group compared to antiplatelet-exposed group in cancer-related stroke patients. Thus, this result should be considered when selecting a secondary prevention drug.

**Supplementary Information:**

The online version contains supplementary material available at 10.1186/s12883-023-03208-4.

## Introduction

Cancer and ischemic stroke are diseases with high morbidity and mortality worldwide [1–3]. The association between these two diseases has been reported frequently. Unlike the traditional stroke etiologies, cerebral infarction in patients with cancer is thought to have a different mechanism, and there are many controversies about it [4–6]. Thromboembolism is a well-known complication affecting 7–18% of cancer patients; thus, cancer-coagulopathy is considered as a potential etiology of ischemic stroke [7, 8]. The mechanism leading to ischemic stroke after cancer coagulopathy has been explained by hypotheses such as the venous system (paradoxical embolism), arteries (intravascular coagulopathy), and heart valves (nonbacterial thrombotic endocarditis, NBTE), and it is known that it can be manifested differently depending on the type, location, extent, and stage of cancer [7, 9, 10].

The treatment guidelines for stroke have already been established, and the worldwide gold standard is set because they are periodically updated and provided by societies such as the American Heart Association/American Stroke Association and European Stroke Organisation [11, 12]. Since the thrombus formation mechanism is different depending on the etiology, the type of antithrombotic agents used after an ischemic stroke attack is also different. For instance, in large artery atherosclerosis or small vessel occlusion, antiplatelet agents are mainly used [12], and in the case of cardioembolism, anticoagulants are generally used [13]. However, in patients with cerebral infarction accompanied by cancer, a gold standard for the use of antithrombotic medications has not been established, and there is controversy about this because the mechanism of action is not clear [14–16]. Currently, it is prescribed according to the judgment of the clinician in clinical practice, but it has not been confirmed which type of antithrombotic drug is more suitable.

In this study, we aimed to investigate the difference of stroke outcomes according to treatments using Korean nationwide claim data.

## Materials and methods

### Data source and study design

In this present study, the National Health Insurance System (NHIS) Customized Research database was used. The database of the National Health Insurance Corporation (NHIC) is a mandatory health insurance system in Korea that includes 52 million (97.3%) of the population in South Korea [17].

Subjects who were enrolled in NHIS during the period between 2010 and 2015 were considered as our study population and were observed until 2019. The data prior to 2010 was used to establish the wash-out period of one year. We settled the index date with the date of stroke diagnosis. During the following dates, death and major bleeding events were checked to compare the anticoagulants group with the antiplatelets group. Subjects previously prescribed antithrombotic medication were excluded. Also, subjects previously diagnosed with major bleeding were excluded. To determine the exact correlation between drug use and mortality, patients who died within 30 days of stroke diagnosis were excluded.

This study was exempted from review by the Yonsei University Health System, Institutional Review Board (Y-2020–0069).

### Operational definitions

The diagnoses in this study were confirmed using the International Classification of Disease 10th edition (ICD-10). The cancer diagnosis was first based on the C00–C97 codes of the ICD-10, and chemotherapy and radiotherapy history were also included to define cancer patients. Procedure codes related to chemotherapy and radiotherapy were selected in consultation with the oncologist, and each code is described in Supplementary materials. To clarify the criteria of cancer, patients who did not receive chemotherapy or radiotherapy were excluded [18]. Seven major types of cancer were selected based on the prevalence and were analyzed separately: Stomach cancer (C16), Colorectal cancer (C18-C20), Liver cancer (C22), Pancreas cancer (C25), Lung cancer (C34), Breast cancer (C50), Prostate cancer (C61).

For the diagnosis of stroke, we developed an appropriate operational definition (because the use of the ICD-10 diagnosis alone is insufficient) as follows: those who had an ICD-10 code of I63 and who underwent computed tomography (CT) or magnetic resonance imaging (MRI) in the brain.

In this study, the first diagnosis dates of stroke and cancer were used to define the cancer-related stroke. Previous studies reported that thromboembolic activity of the active cancer was most powerful within six months before and 12 months after a cancer diagnosis [9, 19]. Patients diagnosed with stroke within the previously described 18 months were defined as cancer-related stroke.

Vascular risk factors used in this study included hypertension (I10-I13, I15), diabetes mellitus (E11-E14), dyslipidemia (E78), coronary artery disease (I20-25), heart failure (I50), atrial fibrillation (I48), valvular heart disease (I34-I39), deep venous thrombosis (I82.9), and pulmonary thromboembolism (I26.9). The detailed operational definitions used in this study are provided in the Supplementary Materials [20, 21].

Because of the policy of NHIS, detailed ingredients of each medication group were not provided, but presented in groups: anticoagulants and antiplatelets. Antiplatelets group included aspirin, clopidogrel, cilostazol, ticlopidine, triflusal, and dipyridamole. Anticoagulants group included warfarin, low molecular weight heparin (dalteparin, enoxaparin, nadroparin, parnaparin, bemiparin, reviparin), and direct oral anticoagulant (apixaban, dabigatran, rivaroxaban, edoxaban).

Major bleeding events as a secondary outcome were defined as a combination of anemia due to acute bleeding, intracranial hemorrhage (ICH), and gastrointestinal (GI) bleeding with ICD-10 codes [20, 22–24]. These detailed operational definitions are also provided in the Supplementary Materials.

### Time-varying exposure [25]

To solve the possible immeasurable time bias because the study was conducted retrospectively based on the previously obtained data, the drug exposure evaluation was conducted by dividing it into daily units. Drug exposure was defined by extracting the cases of prescribed drugs in inpatient and outpatient settings and summing them up for each patient. With a time-varying exposure, each person-day of follow-up was classified as 'exposed to antiplatelet drugs', 'exposed to anticoagulant drugs', 'exposed to both types of drugs', or ‘unexposed to antithrombotic drugs’, and patients could contribute person-time to all the categories. Person-days for which a prescription for the same type of drugs overlapped were duplicated and considered as one type of drug exposure. In this study, both in- and outpatient’s prescription data were used for measuring exposure. In Table 1, to obtain the number of people for each feature according to drug administration in the entire subject, it was defined to exist in only one group per person. On the other hand, in Table 2, each group was defined using the time-varying exposure presented above to obtain the hazard ratio.

**Table 1:** Comparison of baseline characteristics between antithrombotic medication usage groups

	Total (*n* = 2,285)	Antiplatelet only (*n* = 1,829)	Anticoagulant only (*n* = 252)	Combination therapy (*n* = 204)	*P*-value
Age	68.94 ± 10.24	69.05 ± 10.14	67.84 ± 10.86	69.39 ± 10.31	0.174
Age group					
Young age (19–54)	214 (9.37)	169 (9.24)	25 (9.92)	20 (9.80)	0.908
Middle age (55–74)	1,341 (58.69)	1,071 (58.56)	153 (60.71)	117 (57.35)	
Old age (75-)	730 (31.95)	589 (32.20)	74 (29.37)	67 (32.84)	
Sex					
Male	1,581 (69.19)	1,294 (70.75)	149 (59.13)	138 (67.65)	< 0.001
Female	704 (30.81)	535 (29.25)	103 (40.87)	66 (32.35)	
Hypertension	1,563 (68.40)	1,261 (68.94)	169 (67.06)	133 (65.2)	0.490
Diabetes mellitus	633 (27.70)	532 (29.09)	52 (20.63)	49 (24.02)	0.009
Dyslipidemia	601 (26.30)	488 (26.68)	69 (27.38)	44 (21.57)	0.266
Atrial fibrillation	221 (9.67)	65 (3.55)	105 (41.67)	51 (25)	< 0.001
Coronary artery disease	699 (30.59)	538 (29.41)	85 (33.73)	76 (37.25)	0.036
Heart failure	194 (8.49)	130 (7.11)	35 (13.89)	29 (14.22)	< 0.001
Valvular heart disease	28 (1.23)	12 (0.66)	11 (4.37)	5 (2.45)	< 0.001
Deep vein thrombosis	31 (1.36)	23 (1.26)	5 (1.98)	3 (1.47)	0.639
Pulmonary thromboembolism	16 (0.70)	7 (0.38)	6 (2.38)	3 (1.47)	< 0.001
Gastro-intestinal ulcer	1,526 (66.78)	1,237 (67.63)	160 (63.49)	129 (63.24)	0.225
Outcome					
Anemia due to acute bleeding	68 (2.98)	58 (3.17)	5 (1.98)	5 (2.45)	0.523
Intracranial hemorrhage	119 (5.21)	91 (4.98)	17 (6.75)	11 (5.39)	0.491
Gastrointestinal bleeding	671 (29.37)	40 (2.19)	7 (2.78)	7 (3.43)	0.486
Major bleeding	774 (33.87)	56 (3.06)	10 (3.97)	8 (3.92)	0.633
Death	1,531 (67.00)	1,212 (66.27)	185 (73.41)	134 (65.69)	0.071
Cancer type					
Gastric cancer (C16)	276 (12.08)	236 (12.90)	22 (8.73)	18 (8.82)	0.053
Colorectal cancer (C18-20)	346 (15.14)	266 (14.54)	45 (17.86)	35 (17.16)	0.272
Liver cancer (C22)	179 (7.83)	142 (7.76)	19 (7.54)	18 (8.82)	0.852
Pancreas cancer (C25)	101 (4.42)	76 (4.16)	14 (5.56)	11 (5.39)	0.466
Lung cancer (C34)	400 (17.51)	323 (17.66)	41 (16.27)	36 (17.65)	0.861
Breast cancer (C50)	123 (5.38)	95 (5.19)	15 (5.95)	13 (6.37)	0.711
Prostate cancer (C61)	190 (8.32)	153 (8.37)	23 (9.13)	14 (6.86)	0.674

**Table 2:** Comparison of major bleeding and death incidence between drug-exposed types

Unadjusted	*N* (total = 2,285)	Antiplatelet	Anticoagulation	Combination	Discontinuation
Intracranial hemorrhage	119 (5.21)	1	1.800 (1.433—2.259)	1.798 (0.928—3.484)	0.672 (0.584—0.774)
Gastrointestinal bleeding	671 (29.37)	1	1.196 (1.062—1.345)	1.755 (1.293—2.383)	0.684 (0.642—0.728)
Major bleeding	774 (33.87)	1	1.326 (1.189—1.480)	1.639 (1.207—2.225)	0.707 (0.665—0.750)
Death	1,531 (67.00)	1	1.127 (1.056—1.204)	1.912 (1.640—2.229)	1.155 (1.117—1.194)
Model 1^a^	*N* (total = 2,285)	Antiplatelet	Anticoagulation	Combination	Discontinuation
Intracranial hemorrhage	119 (5.21)	1	1.826 (1.453—2.294)	1.943 (1.002—3.767)	0.689 (0.598—0.792)
Gastrointestinal bleeding	671 (29.37)	1	1.214 (1.078—1.366)	1.710 (1.259—2.321)	0.689 (0.647—0.733)
Major bleeding	774 (33.87)	1	1.348 (1.208—1.504)	1.619 (1.192—2.198)	0.712 (0.671—0.756)
Death	1,531 (67.00)	1	1.140 (1.068—1.218)	1.771 (1.519—2.065)	1.181 (1.142—1.221)
Model 2^b^	*N* (total = 2,285)	Antiplatelet	Anticoagulation	Combination	Discontinuation
Intracranial hemorrhage	119 (5.21)	1	1.427 (1.104—1.845)	1.569 (0.805—3.056)	0.659 (0.572—0.759)
Gastrointestinal bleeding	671 (29.37)	1	1.284 (1.129—1.460)	1.709 (1.256—2.325)	0.688 (0.646—0.732)
Major bleeding	774 (33.87)	1	1.350 (1.198—1.522)	1.537 (1.130—2.091)	0.706 (0.665—0.750)
Death	1,531 (67.00)	1	1.041 (0.968—1.119)	1.715 (1.469—2.002)	1.188 (1.149—1.229)

^a^ Age, sex adjusted

^b^ Age, sex, hypertension, diabetes mellitus, dyslipidemia, atrial fibrillation, coronary artery disease, heart failure, valvular heart disease, deep venous thrombosis, pulmonary thromboembolism, and gastrointestinal ulcer adjusted

### Statistical analysis

The one-way analysis of variance (ANOVA) was used for comparing the continuous variables among the groups, while Pearson χ2 test was used for comparing categorical variables.

The study exposure was antiplatelet drugs, considered as a time-varying variable. We calculated hazard ratios (HRs) with 95% confidence intervals for outcomes using a Cox proportional hazards regression model with time-dependent covariates.

We used 2 models with increasing degrees of adjustment to account for potential confounding factors at baseline. Model 1 was adjusted for age and sex. Model 2 was further adjusted for hypertension, diabetes mellitus, dyslipidemia, coronary artery disease, heart failure, atrial fibrillation, deep venous thrombosis, pulmonary thromboembolism, and gastrointestinal ulcer.

All statistical analyses were performed with SAS version 9.4 (SAS Institute, Cary, NC, USA). A 2-sided *p* value of < 0.05 was generally considered a minimum level of statistical significance.

## Results

**Fig. 1:**
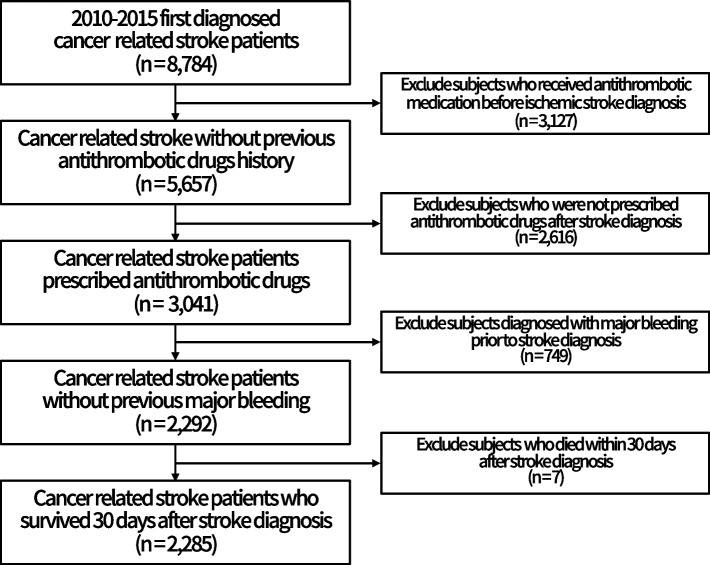
Selection of study subjects from the National Health Insurance Service National Sample Cohort database of South Korea

Between January 1, 2010, and December 31, 2015, 51,456 subjects 19 years of age or older who meet the operational definition of ischemic stroke were included in the NHIS database. As mentioned in the methods section, a total of 8,784 subjects were defined as cancer-related stroke by considering the period between diagnosis of cancer and cerebral infarction. According to the exclusion criteria presented above, subjects who received antithrombotic medication before ischemic stroke diagnosis (*n* = 3,127) and patients who were not prescribed antithrombotics after stroke diagnosis (*n* = 2,616) were excluded. In addition, patients diagnosed with major bleeding prior to stroke diagnosis (*n* = 749) and patients who died within 30 days after stroke diagnosis (*n* = 7) were also excluded. Finally, a total of 2,285 subjects remained in the analysis (Fig. 1).

### Demographic characteristics

The baseline demographic characteristics according to the type of medicine are presented in Table 1. Of the total 2,285 subjects, 1,829 (80.0%) were prescribed only antiplatelet drugs during the follow-up period, 252 (11.0%) were prescribed only anticoagulant drugs, and 204 (8.9%) had a record of receiving both types of antithrombotic drugs. Differences in age, sex, comorbidity, cancer type, and outcome were compared and analyzed between the three groups. There were significant differences in sex and some comorbidities (dyslipidemia, atrial fibrillation, coronary artery disease, heart failure, valvular heart disease, pulmonary thromboembolism). These differences between groups were prominent in cardiac disease, and the proportion of patients in the anticoagulant and combination group was higher.

### Comparison of major bleeding and death incidence between groups

Table 2 shows the estimated hazard ratios (HRs) and 95% confidence intervals (CIs) for all causes of mortality and major bleeding events associated with cancer-related stroke patients' current prescription status. During the follow-up period, 1,531 (67.0%) deaths occurred, and major bleeding occurred in 774 (33.9%) patients, of which 119 (5.2%) had an intracranial hemorrhage and 671 (29.4%) had gastrointestinal bleeding.

A group with anticoagulation showed high HRs of all bleeding events in multivariate analyses (ICH HR, 1.43; 95% CI, 1.10–1.85; *p* = 0.007, GI bleeding HR, 1.28; 95% CI, 1.13–1.46; *p* < 0.001, major bleeding HR, 1.35; 95% CI, 1.20–1.52; *p* < 0.001). And the result was also similar in the combination group (ICH HR, 1.57; 95% CI, 0.81–3.06; *p* = 0.186, GI bleeding HR, 1.71; 95% CI, 1.26—2.33; *p* < 0.001, major bleeding HR, 1.54; 95% CI, 1.13–2.09; *p* = 0.006). Based on the antiplatelet group, the hazard ratio of bleeding occurrence in the target group was significant overall groups except HR of intracranial hemorrhage occurrence in the combination group. Compared to antiplatelet group, discontinuation group showed significantly low HRs of all bleeding events in multivariate analyses (Model 2. ICH HR, 0.66; 95% CI, 0.57–0.76; *p* < 0.001, GI bleeding HR, 0.69; 95% CI, 0.65–0.73; *p* < 0.001, major bleeding HR, 0.71; 95% CI, 0.67–0.75; *p* < 0.001).

**Fig. 2:**
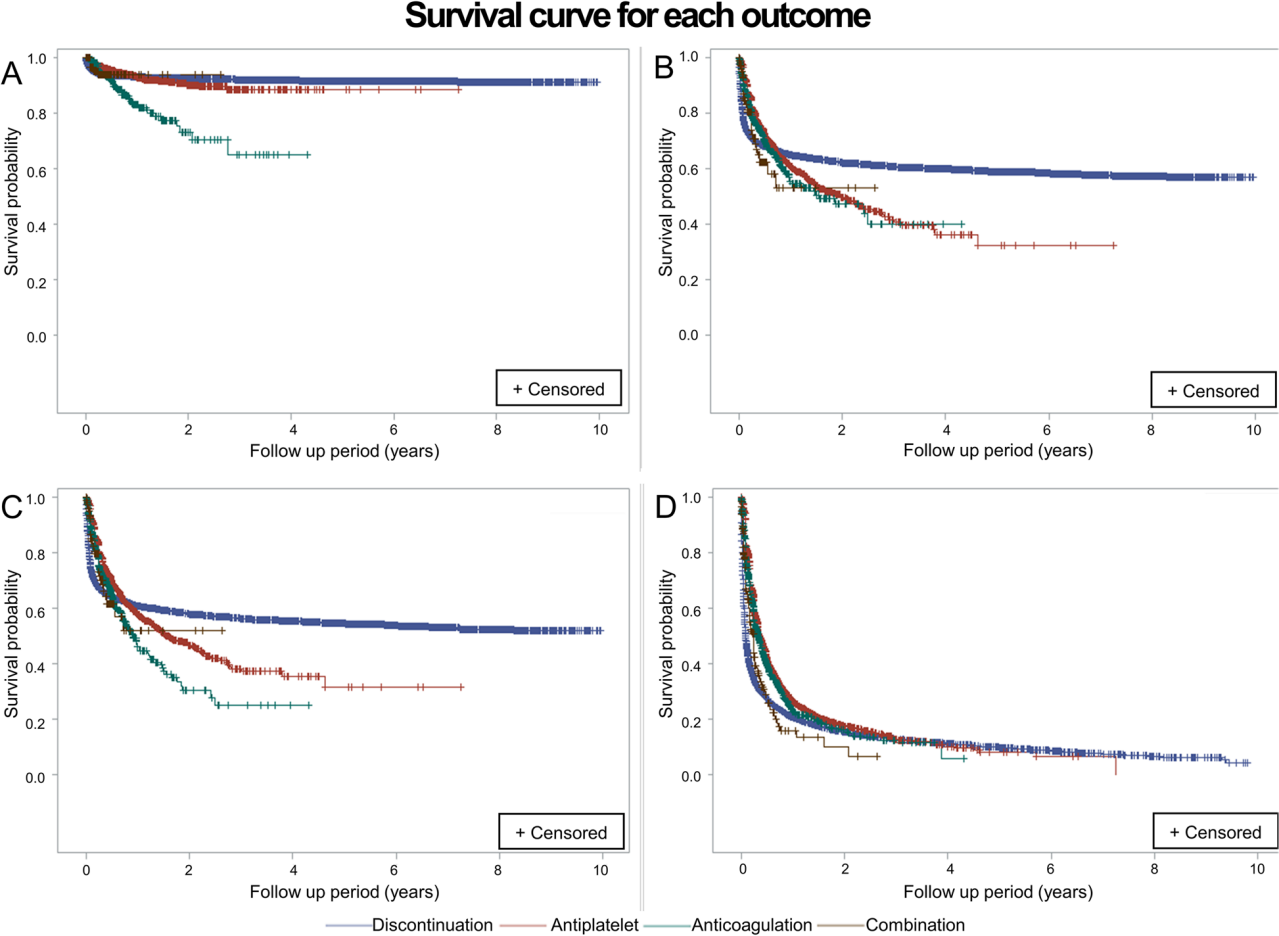
The occurrence of intracranial hemorrhage **A**, gastrointestinal bleeding **B**, major bleeding **C**, death **D** among cancer-related stroke patients: a retrospective observational cohort study in Korea

Figure 2 shows each group's survival plots for bleeding events and death. The discontinuation group showed significant mortality HRs (HR, 1.19; 95% CI, 1.15–1.23; *p* < 0.001), and this was similar to the combination group (HR, 1.72; 95% CI, 1.47–2.00; *p* < 0.001). A group with anticoagulation also showed high mortality HRs compared to the antiplatelet group (HR, 1.04; 95% CI, 0.97–1.12; *p* = 0.282), while this result was statistically insignificant.

### Subgroup analyses with demographic features, comorbidities, and cancer types

**Fig. 3:**
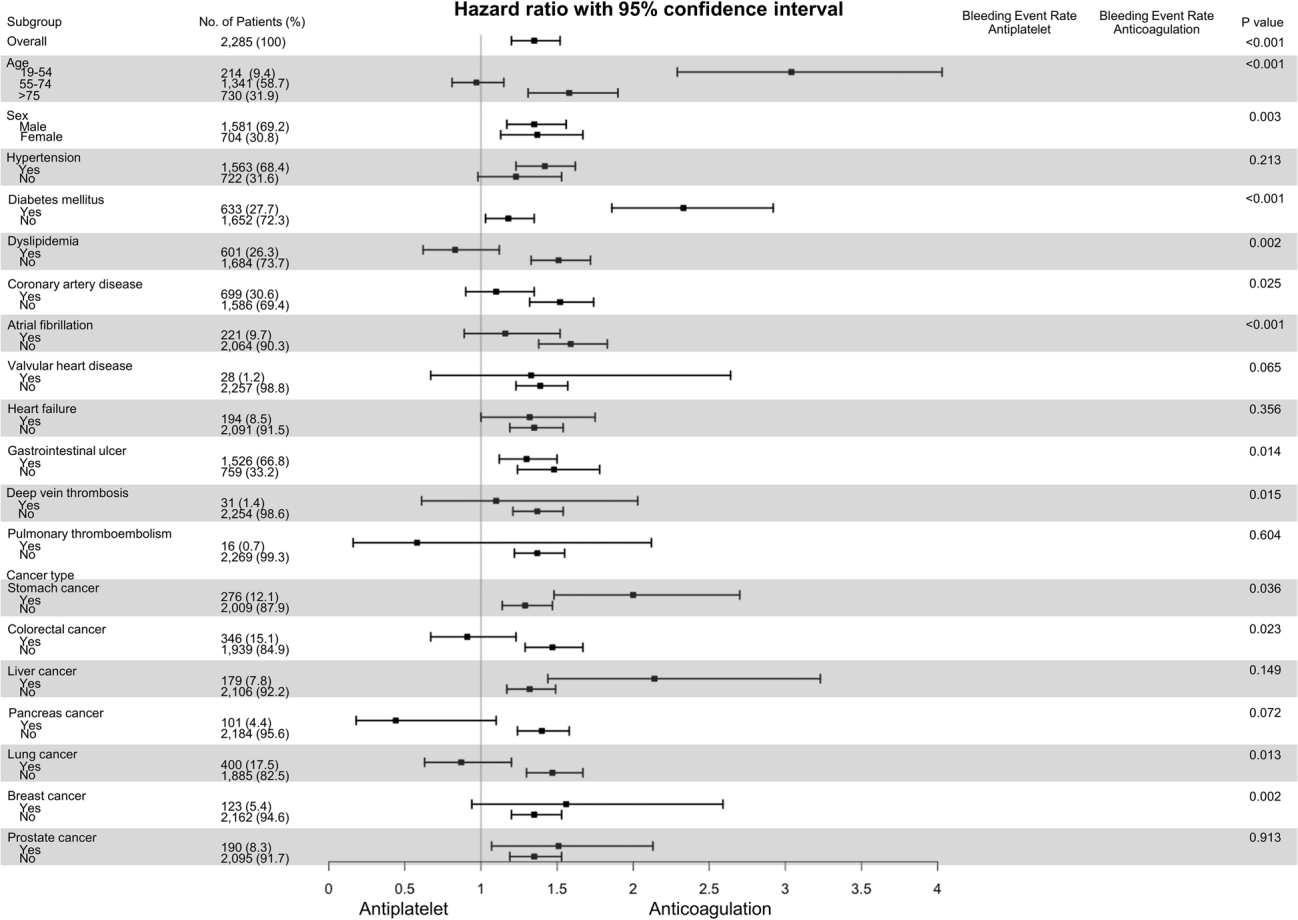
Forest plot for a hazard ratio with 95% confidence interval of the major bleeding events when using anticoagulants compared to antiplatelets in the subgroup analysis

Subgroup analysis was done by dividing subjects into young age (19–54 years old), middle-age (55–74 years old), and old age (> 74 years old) [26], and it was confirmed that the young age group showed particularly high HR of the major bleeding events when using anticoagulants compared to antiplatelets only. There were significant differences with subgroup analyses with some comorbidities. Patients who had diabetes mellitus showed higher HR of the major bleeding events than patients without diabetes. And this may affect higher mortality HR with diabetes mellitus, compared to those without the disease. However, other comorbidities showed different trends. Patients with dyslipidemia, coronary artery disease, atrial fibrillation, or deep venous thrombosis showed lower HR in major bleeding events than those without (Fig. 3 and Supplementary Table 4).

Seven types of cancer (lung, colorectal, stomach, prostate, liver, breast, pancreas) were selected in the order of highest frequency, and subgroup analysis was performed on each. Subjects with stomach (ICD-10 codes, C16), liver (C22), breast (C50), or prostate (C61) cancer showed higher HRs of major bleeding events in the 'exposed to anticoagulant drugs' group compared to other types of cancer. On the other side, subjects with colorectal (C18-20), pancreas (C25), or lung (C34) cancer showed lower HRs of major bleeding events in the 'exposed to anticoagulant drugs' group compared to other types of cancer patients.

## Discussion

In this study, the risk of major bleeding events and deaths were compared by antithrombotic drug exposure type. Among the 'cancer-related stroke’ patients defined by considering the duration, the risk of all types of bleeding was observed to increase in the anticoagulant-exposed group compared to the antiplatelet-exposed group, and this result was corresponded even after adjusting for age, sex, and other comorbidities related to stroke.

In previous studies, the bleeding risk was compared between the antiplatelet and anticoagulation groups. According to a previously published systematic review paper, in the anticoagulation therapy group with nonrheumatic atrial fibrillation, the risk of recurrent stroke is lower, the risk of major extracranial bleeding is higher, and the risk of intracranial hemorrhage is not affected compared to antiplatelet [27]. Other researchers published systematic review and meta-analysis of two drug groups in elderly patients with atrial fibrillation, and they included seven RCTs and four cohort studies [28]. They reported that the risk of major bleeding between the antiplatelet-treated group and the anticoagulation-treated group was equal, but the risk of all types of bleeding and intracerebral hemorrhage was lower in the antiplatelet-treated group. Moreover, many studies have reported that the risk of bleeding significantly increases when antiplatelet and anticoagulant are treated simultaneously. Through this paper, it was inferred that the risk of major bleeding of all types was significantly increased when anticoagulation was administered in the cancer-related stroke patient group and even higher when combination therapy was done.

The risk of mortality, in contrast, showed similar but slightly different results compared to the risk of bleeding events. Increased mortality risk was observed in all types of exposure groups (even in ‘unexposed to antithrombotic drugs’ group) compared to antiplatelet-exposed group. However, mortality risk in the anticoagulant-exposed group was not statistically significant in multivariate analysis with model 2 that included age, sex, and other comorbidities related to stroke. Clinically vitally unstable cancer patients often do not receive antithrombotic therapies for secondary prevention of ischemic stroke for fear of increased bleeding risk, which could explain the high mortality in the non-exposed group compared to the antiplatelet group. Even considering such a point, it was possible to confirm that the combination group showed a high mortality risk compared to other groups, and additional analysis was performed to verify the association between bleeding events and death. The average survival time after major bleeding events was 681.95 days (standard deviation, 755.75 days), and there was a significant relationship between bleeding events and death (mortality HR, 1.07; 95% CI, 1.04–1.11; *p* < 0.001).

Subgroup analysis showed that a major bleeding risk was significantly increased in the young age group with anticoagulation. This may be because stroke etiology is unique in the young age group. In the absence of comorbidities that usually require anticoagulation therapy, an increased risk of bleeding occurrence was also observed when anticoagulation treatment was performed compared to antiplatelet treatment. From this, it can be inferred that the use of anticoagulants may enhance the bleeding risk in cancer-related stroke patients, where the mechanism of thrombus formation is not clearly visible. This phenomenon may have occurred due to better management of the warfarin titer in other diseases requiring anticoagulant administration. Since there are not many methods to evaluate the influence of cancer itself in the Korean nationwide claim data, subgroup analysis was performed according to cancer type for minimal correction. Subjects with stomach, liver, breast, or prostate cancer showed a higher risk of major bleeding events when using anticoagulants, while subjects with colorectal, pancreas, or lung cancer showed the opposite results. This was different from a previous study which measured cancer patients’ bleeding risk within six months of starting anticoagulation per cancer types [29]. They reported that cancer with higher Khorana risk score and lumen gastrointestinal cancer patients had higher bleeding risks compared to others. This difference may occur because our study measured lifelong bleeding events, and we included only advanced cancer patients.

### Limitations

There are several limitations of this study. First, due to the nature of the dataset, this study was performed with retrospective observational cohort studies. Therefore, results cannot provide causal relationships, and the conclusions reflect associations that should not be overstated. There might be remaining possibilities for confounding factors, though we tried to minimize that with multivariate analysis. Second, since the NHIC database does not offer any laboratory data and image data, the quality of anticoagulation control among those prescribed warfarin and stroke severity that helps determine the risk of intracranial hemorrhage cannot be evaluated. Also, other laboratory data which are related to bleeding tendency cannot be assessed. Due to the characteristics of the claim database, detailed clinical information such as stroke severity and cancer stage. The characteristics of cancer itself can affect bleeding risk or mortality. Still, since information about this cannot be obtained, the definition of cancer patients is limited to those who have received chemotherapy or radiation therapy. Through this, it can be estimated that the cancer patients identified in this study have advanced cancers to some extent. Third, some inaccurate definitions may have been made in the operational definition process. Especially, we defined cancer-related ischemic stroke using the period between cancer and the initial diagnosis of cerebral infarction, and stroke caused by etiology other than those caused by active cancer effect may be included. Moreover, advanced cancer was defined with chemotherapy and radiotherapy without surgery information, and this may lead to loss of some types of cancer patients. Fourth, stroke recurrence risk was usually checked as the primary outcome in the study of secondary prevention drugs, but it was difficult to define it with a NIHC claim database, so it was not possible to proceed. Instead, we mainly identified the side effects of the drug by measuring bleeding risk and mortality. Last, since this study primarily focused on Asians, extrapolation of the study results to non-Asian cancer-related stroke patients would be cautiously managed.

## Conclusions

In most cases of cancer-related stroke patients, it was verified that the bleeding risk increased in the order of antiplatelet-exposed group, anticoagulant-exposed group, and both types of antithrombotic-exposed group. It is thought that it is necessary to consider this risk when selecting a secondary prevention drug, and it would be good if further studies on the mechanism were made based on these results.

## Supplementary Information

**Additional file 1. **Supplementary materials.

## Data Availability

All data are available from the database of National Health Insurance Sharing Service (NHISS) https://nhiss.nhis.or.kr/. NHISS allows access to all of this data for the any researcher who promises to follow the research ethics at some cost. If you want to access the data of this article, you can download it from the website after promising to follow the research ethics. Releasing of the data by the researcher is not allowed legally. Releasing of the data by the researcher is not allowed legally.
