# Correction: Bleeding risk and mortality according to antithrombotic agents’ exposure in cancer-related stroke patients: nationwide population-based cohort study in South Korea

**DOI:** 10.1186/s12883-023-03247-x

**Published:** 2023-05-19

**Authors:** Bo Kyu Choi, Ji Sung Lee, Hae Reong Kim, Han Sang Kim, Yo Han Jung, Yu Rang Park

**Affiliations:** 1 Department of Biomedical Systems Informatics, Yonsei University College of Medicine, Seoul, Republic of Korea; 2 Clinical Research Center, Asan Medical Center, Asan Institute for Life Sciences, University of Ulsan College of Medicine, Seoul, 05505 Korea; 3 Yonsei Cancer Center, Division of Medical Oncology, Department of Internal Medicine, Yonsei University College of Medicine, Seoul, Korea; 4 Graduate School of Medical Science, Severance Biomedical Science Institute, Brain Korea 21 Project, Yonsei University College of Medicine, Seoul, Korea; 5 Department of Neurology, Gangnam Severance Hospital, Yonsei University College of Medicine, Seoul, Korea

**Correction: BMC Neurol 23, 187 (2023)**

10.1186/s12883-023-03208-4

Following publication of the original article [1], the authors reported an error in Funding section. The original article [1] states “Not applicable” in the Funding section. However, since the authors received financial support from the Ministry of Trade, Industry & Energy (MOTIE, Korea) through the Bio-Industrial Technology Development Program (20014841), it is necessary to add the following statement to the Funding section: “This work was supported by the Bio-Industrial Technology Development Program (20014841) funded by the Ministry of Trade, Industry & Energy (MOTIE, Korea).”

The original article [1] has been updated.
