# Supplementary material for: Bleeding risk and mortality according to antithrombotic agents’ exposure in cancer-related stroke patients: nationwide population-based cohort study in South Korea
Source: BMC Neurol. 2023 May 9;23:187. doi: 10.1186/s12883-023-03208-4 (PMC10169453; doi:10.1186/s12883-023-03208-4)
Supplement: Supplementary file 1 — Additional file 1. Supplementary materials. [file 12883_2023_3208_MOESM1_ESM.docx]

eTable 1. Operational definitions

| Diagnosis | ICD-10-CM & Definition |
| --- | --- |
| Cancer | C00-97 & radiotherapy/chemotherapy |
| Stroke | I63 & brain CT/MRI |
| Cancer related stroke | Patients diagnosed with stroke 6 months before or 12 months after the cancer diagnosis |
| Atrial fibrillation | I48 |
| Coronary artery disease | I20-I25 |
| Heart failure | I50 |
| Valvular heart disease | I34-I39 |
| Deep vein thrombosis | I82.9 |
| Pulmonary thromboembolism | I26.9 |
| Gastro-intestinal ulcer | K25-K28 |
| Hypertension | I10-I13, I15 & subjects who were prescribed anti-hypertensive medication |
| Diabetes mellitus | E11-E14 & Subjects who were prescribed diabetes medication |
| Dyslipidemia | E78 & Subjects who were prescribed statin |
|  |  |
| Procedure |  |
| Radiotherapy (procedure codes) | HD056, HD053, HD061, HD055, HD052, HZ271, HD081, HD085, HD072, HD071, HA416, HA443, HA441, HA445, HA447, HA446, HA448, HA444, HD015, HD415, HD419, HD022, HD013, HD413, HD014, HD414, HD041, HD441, HD018, HD418, HD019, HD010, HD410, HD011, HD411, HD092, HD091, HY402, HY404, HD113, HD114, HD211, HD111, HD212, HD033, HD031, HD032, HX401, HD112, HD093, HY405, HD023 |
| Chemotherapy (Ingredient codes) | 620402ATB, 626101ATB, 626102ATB, 626103ATB, 658801BIJ, 658802BIJ, 503701BIJ, 104501BIJ, 656201ACH, 358301ACH, 108801BIJ, 109001ATB, 588201ATB, 588202ATB, 588203ATB, 588204ATB, 588205ATB, 588206ATB, 588207ATB, 588430BIJ, 657701BIJ, 621001ATB, 621002ATB, 484301BIJ, 484302BIJ, 452801BIJ, 614602BIJ, 614601BIJ, 554330BIJ, 554331BIJ, 117201ATB, 117202ATB, 118001BIJ, 647701BIJ, 463303BIJ, 463301BIJ, 624501BIJ, 675701ATB, 120401CSI, 120602BIJ, 120630BIJ, 120601ATB, 613901BIJ, 666403ATB, 666402ATB, 666401ATB, 122701ATB, 122702ATB, 123730BIJ, 123731BIJ, 123732BIJ, 123733BIJ, 123735BIJ, 123734BIJ, 647802BIJ, 647801BIJ, 124301ATB, 634401ACH, 556401BIJ, 556430BIJ, 130901ATB, 134503BIJ, 134501BIJ, 134530BIJ, 134533BIJ, 134534BIJ, 134830BIJ, 614330BIJ, 617501ACH, 617502ACH, 139005BIJ, 139003BIJ, 139001ATB, 139004BIJ, 194701ACS, 194702ACS, 139601BIJ, 139604BIJ, 139602BIJ, 139605BIJ, 139634BIJ, 139635BIJ, 139603BIJ, 139631BIJ, 139632BIJ, 139636BIJ, 139638BIJ, 139637BIJ, 139633BIJ, 663101ACH, 663102ACH, 139901BIJ, 139902BIJ, 140101BIJ, 667101BIJ, 667102BIJ, 493301ATB, 493302ATB, 493303ATB, 493304ATB, 493305ATB, 140601BIJ, 495601BIJ, 495602BIJ, 624402BIJ, 624401BIJ, 364701ATB, 148341BIJ, 148342BIJ, 148345BIJ, 148349BIJ, 148344BIJ, 148348BIJ, 148301BIJ, 148302BIJ, 148346BIJ, 148350BIJ, 148351BIJ, 149301ACH, 149302ACH, 149402BIJ, 149401BIJ, 149430BIJ, 149432BIJ, 149433BIJ, 151901BIJ, 627401ACS, 152701BIJ, 152702BIJ, 152730BIJ, 152731BIJ, 621330BIJ, 477401ATB, 477402ATB, 477403ATB, 155101ACH, 157101BIJ, 157131BIJ, 157132BIJ, 157102ACH, 485606ATB, 485607ATB, 485605ATB, 358401ATB, 158933BIJ, 158903BIJ, 158930BIJ, 158931BIJ, 158936BIJ, 158934BIJ, 158935BIJ, 160101BIJ, 160102ATB, 161401BIJ, 161402BIJ, 161430BIJ, 161431BIJ, 161432BIJ, 568931BIJ, 453001ATB, 164930BIJ, 164931BIJ, 164903BIJ, 164932BIJ, 167202BIJ, 167201BIJ, 168801BIJ, 168802BIJ, 172001ACH, 172002ACH, 628101ACH, 173002BIJ, 173002ACH, 173301BIJ, 412701ATB, 412703ATB, 412702ATB, 412704ATB, 175504BIJ, 175502BIJ, 175530BIJ, 175601BIJ, 175630BIJ, 175631BIJ, 175602BIJ, 177430BIJ, 177431BIJ, 177433BIJ, 177435BIJ, 507501ATB, 181401BIJ, 181403BIJ, 588201ACH, 588202ACH, 588205ACH, 588203ACH, 588204ACH, 588206ACH, 588207ACH, 645202ACH, 645201ACH, 182201ATB, 182602BIJ, 182601BIJ, 182630BIJ, 182604BIJ, 182610BIJ, 182611BIJ, 182606BIJ, 182608BIJ, 182605BIJ, 149431BIJ, 149435BIJ, 188905ATB, 189301ASS, 189330ASS, 189331ASS, 189304ASS, 189335ASS, 189301ATB, 189302ATB, 189303ASS, 189902BIJ, 189901ATB, 190601ATB, 192104BIJ, 192102BIJ, 192103BIJ, 192105BIJ, 192139BIJ, 192141BIJ, 192142BIJ, 192143BIJ, 192101ATB, 196401BIJ, 196402BIJ, 196501BIJ, 196530BIJ, 196502BIJ, 562602ACH, 562601ACH, 202101BIJ, 638401BIJ, 638402BIJ, 628901BIJ, 643501ACH, 658901BIJ, 652501ATB, 652502ATB, 205801BIJ, 205802BIJ, 205803BIJ, 205830BIJ, 205834BIJ, 205832BIJ, 207801BIJ, 207802BIJ, 207830BIJ, 207831BIJ, 207804BIJ, 207832BIJ, 207835BIJ, 207833BIJ, 207805BIJ, 655203ACH, 655202ACH, 655201ACH, 611801ATB, 611802ATB, 639001BIJ, 481203BIJ, 481239BIJ, 481233BIJ, 481234BIJ, 481232BIJ, 481230BIJ, 481231BIJ, 624601BIJ, 100101APD, 100102ACH, 100130ALQ, 213502BIJ, 215330AGN, 215301ACH, 628002ACH, 628003ACH, 628004ACH, 669601ATB, 669602ATB, 174301BIJ, 617701ACH, 617702ACH, 639301BIJ, 639302BIJ, 624801ATB, 422630BIJ, 422631BIJ, 422603BIJ, 422632BIJ, 623002ATB, 623003ATB, 623001ATB, 100201BIJ, 100202BIJ, 647901BIJ, 647902BIJ, 228101BIJ, 228130BIJ, 488001ATB, 231902BIJ, 487701ACH, 487702ACH, 487703ACH, 234203ACH, 234201ACH, 234204ACH, 234501ATB, 234502ATB, 234801ACH, 358203ACH, 358202ACH, 358204ACH, 568230BIJ, 485701ACH, 485702ACH, 237901ATB, 198001BIJ, 198003BIJ, 241901BIJ, 241902ACH, 241903ACH, 242101ATB, 645402ATB, 645401ATB, 242802BIJ, 242801BIJ, 242830BIJ, 626001BIJ, 626002BIJ, 243001ACS, 244902BIJ, 244901BIJ, 244930BIJ, 467502BIJ, 467501BIJ, 388501ACH, 388502ACH, 624101ATB, 624102ATB, 620501ATB, 247801BIJ, 247830BIJ, 248001BIJ, 248003BIJ, 248030BIJ, 248031BIJ, 248201BIJ, 248202BIJ, 248230BIJ, 248231BIJ, 248504BIJ, 248505BIJ, 248507BIJ, 248506BIJ, 248502BIJ, 248503BIJ, 248508BIJ, 248509BIJ, 248510BIJ, 248604BIJ, 248605BIJ, 248607BIJ, 248606BIJ, 248601BIJ, 248602BIJ, 248603BIJ, 309700ACH, 309600AGN, 364100AGN, 309600ACE, 452400ACH, 452500ACH, 164602BIJ, 123702BIJ, 123701BIJ, 123703BIJ, B0085801, 192106BIJ, 123704BIJ, 161403BIJ, 164630COO, 248101BIJ, 149404BIJ, 149405BIJ, 192138BIJ, 192140BIJ, 134502BIJ, 134532BIJ, 157104BIJ, 157105BIJ, 157107BIJ, 157108BIJ, B0219601, B0220201, 139903BIJ, 248002BIJ, 157103BIJ, 157106BIJ, 157135BIJ, 161404BIJ, 139609BIJ, 139630BIJ, 149403BIJ, 149406BIJ, 248032BIJ, B1035701, 134531BIJ, B0421001, 177401BIJ, 177402BIJ, 164902BIJ, 164901BIJ, 123707BIJ, 123708BIJ, 148306BIJ, 148309BIJ, 148310BIJ, 481201BIJ, 177408BIJ, 177437BIJ, 148304BIJ, 422601BIJ, 422602BIJ, 481202BIJ, 481205BIJ, 205804BIJ, 177403BIJ, 177404BIJ, 177407BIJ, 177436BIJ, 207803BIJ, 149434BIJ, 157130BIJ, 157133BIJ, 157134BIJ, 157136BIJ, 152703BIJ, 152704BIJ, 152732BIJ, 152733BIJ, 123706BIJ, 123736BIJ, 554301BIJ, 554302BIJ, 196403BIJ, 556402BIJ, 556431BIJ, 614301BIJ, 215302AGN, 205805BIJ, 205833BIJ, 177406BIJ, 177434BIJ, 177405BIJ, 588401BIJ, 481204BIJ, 481237BIJ, 477801ATB, 242803BIJ, 189336ASS, 182609BIJ, 189333ASS, 189337ASS, 189338ASS, 189334ASS, 182607BIJ, 158902BIJ, 158901BIJ, 175607BIJ, 175608BIJ, 175603BIJ, 175604BIJ, 175605BIJ, 175606BIJ, 100101ALQ, 192107ATB, 311500ATB |
| Brain CT (procedure codes) | HA451XXX, HA441XXX, HA461XXX, HA471XXX, HA851XXX |
| Brain MRI (procedure codes) | HE101XXX, HE135XXX, HE136XXX, HE201XXX, HE235XXX, HE236XXX, HE301XXX, HE401XXX, HE501XXX, HE535XXX, HE536XXX, HF101XXX, HF201XXX, HF202XXX |
|  |  |
| Medication |  |
| Antiplatelet | aspirin, clopidogrel, cilostazol, ticagrelor, ticlopidine, triflusal, and dipyridamole |
| Anticoagulant | warfarin, low molecular weight heparin (dalteparin, enoxaparin, nadroparin, parnaparin, bemiparin, reviparin), and direct oral anticoagulant (apixaban, dabigatran, rivaroxaban, edoxaban) |
| Antihypertensive drugs (Ingredient codes) | 104201ATB, 104202ATB, 107601ATB, 107602ATB, 107603ATB, 111402ATB, 111403ATB, 114001ACH, 114002ACH, 114003ACH, 115101ATB, 115102ATB, 115103ATB, 115104ATB, 116801ATB, 116803ATB, 117001ATB, 117002ATB, 122601ATB, 122602ATB, 122603ATB, 122901ATB, 122902ATB, 122903ATB, 125001ATB, 125002ATB, 125003ATB, 125004ACR, 125005ATB, 125006ACR, 125007ACR, 125007ATR, 125008ACR, 125008ATR, 133001ATB, 133002ATB, 133003ATB, 133101ATB, 133102ATB, 149101ATB, 149102ATB, 149104ATR, 151601ATB, 151603ATB, 157501ATR, 157503ATR, 170701ATB, 174401ATR, 177301ATB, 177303ATB, 180301ATB, 180302ATB, 180303ATB, 182001ATB, 184501ATB, 185701ATB, 185702ATB, 185703ATB, 188001ATB, 188002ATB, 196102ATB, 201003ACR, 211301ATB, 211302ATB, 222401ATB, 222402ATB, 222404ATB, 247101ATB, 247102ATB, 247103ATB, 247104ATB, 262100ATB, 262400ATR, 262500ATB, 378900ATB, 486900ATB, 262700ATB, 356400ATB, 442600ATB, 378801ATB, 378802ATB, 378803ATB, 385700ATB, 385800ATB, 423700ATB, 429201ATB, 443200ATB, 443300ATB, 502600ATB, 447100ATB, 447200ATB, 459801ACH, 459801ATB, 459802ACH, 459901ATB, 459902ATB, 460500ATB, 464601ATB, 468501ATB, 468502ATB, 468503ATB, 469800ATB, 469900ATB, 470000ATB, 470801ATB, 470802ATB, 476201ATB, 479701ATB, 483101ATB, 483102ATB, 483201ATB, 483202ATB, 483401ACH, 485201ATB, 485202ATB, 486501ATB, 486502ATB, 486503ATB, 489501ATB, 489502ATB, 489503ATB, 492800ATB, 492900ATB, 495800ATB, 500500ATB, 582200ATB, 582400ATB, 501601ATB, 501602ATB, 502700ATB, 503000ATB, 513900ATB, 510401ATB, 510402ATB, 510403ATB, 511500ATB, 511600ATB, 511700ATB, 623100ATB, 513600ATB, 515201ATB, 515202ATB, 515203ATB, 519700ATB, 519800ATB, 520000ATB, 521200ATB, 521300ATB, 521400ATB, 644800ATB, 522000ATB, 526800ATB, 522200ATB, 522300ATB, 522400ATB, 522600ATB, 522700ATB, 522800ATB, 522900ATB, 523000ATB, 523100ATB, 523200ATB, 523300ATB, 523400ATB, 547600ATB, 547700ATB, 547800ATB, 547900ATB, 548000ATB, 631300ATB, 553301ATB, 556200ATB, 564701ATB, 564702ATB, 629400ATB, 629500ATB, 629600ATB, 632201ATB, 632800ATB, 632900ATB, 633000ATB, 637400ATB, 637500ATB, 637600ATB, 651900ATB, 652000ATB, 652100ATB, 652700ATB, 652301ATB, 652302ATB, 652303ATB, 652900ATB, 653000ATB, 653100ATB, 662201ATB, 662401ATB, 662402ATB, 662403ATB, 662800ATB, 662900ATB, 663000ATB, 663500ATB, 663600ATB, 663700ATB, 663800ATB, 673500ATB, 673600ATB, 682700ATB, 682800ATB, 682900ATB, 697300ATB, 697400ATB, 697500ATB, 697600ATB |
| Diabetes medicine (Ingredient codes) | 100601ATB, 100602ATB, 165402ATB, 165602ATB, 165603ATR, 165604ATR, 165701ATB, 165702ATB, 165703ATB, 165704ATB, 165801ATB, 191501ATB, 191502ATB, 191502ATR, 191503ATB, 191504ATB, 191504ATR, 191505ATR, 249001ATB, 249002ATB, 379501ATB, 379502ATB, 379503ATB, 430201ATB, 430202ATB, 430203ATB, 431901ATB, 431902ATB, 443400ATB, 443500ATB, 474200ATB, 474300ATB, 474300ATR, 498600ATB, 486101ATB, 497200ATB, 498100ATB, 500801ATB, 501101ATB, 501102ATB, 501103ATB, 502300ATB, 502300ATR, 502900ATB, 513700ATB, 513700ATR, 524700ATR, 507000ATB, 507100ATB, 519600ATB, 518500ATR, 518600ATR, 518800ATB, 520500ATB, 520600ATB, 520700ATB, 523800ATR, 632000ATR, 645000ATR, 654100ATR, 525500ATB, 525600ATB, 525901ATB, 527302ATB, 613301ATB, 613302ATB, 616401ATB, 619101ATB, 624202ATB, 624203ATB, 627301ATB, 628201ATB, 628202ATB, 630500ATB, 630600ATB, 635600ATB, 635700ATB, 675500ATB, 636101ATB, 639601ATB, 639800ATR, 641400ATR, 641800ATR, 641900ATR, 642000ATR, 645301ATB, 648400ATB, 648500ATB, 648600ATB, 649000ATB, 649100ATB, 649200ATB, 649300ATB, 649400ATB, 649500ATB, 649900ATR, 650000ATR, 650100ATR, 653800ATR, 653900ATR, 654000ATR, 655700ATR, 664600ATB, 664700ATB, 664800ATB, 671800ATR, 673800ATR, 671900ATR, 672000ATR, 672100ATR, 672500ATR, 672600ATR, 672700ATR, 672800ATR, 672900ATR, 683300ATR, 683400ATR, 674301ATB, 674302ATB, 170130BIJ, 170131BIJ, 170430BIJ, 170431BIJ, 175330BIJ, 175331BIJ, 175332BIJ, 175333BIJ, 441330BIJ, 441331BIJ, 441332BIJ, 441333BIJ, 461830BIJ, 461831BIJ, 461832BIJ, 484930BIJ, 484931BIJ, 488730BIJ, 512130BIJ, 512131BIJ, 626700BIJ, 626830BIJ, 639701BIJ, 639702BIJ, 666700BIJ, 667000BIJ, 693900BIJ |
| Statin | atorvastatin, fenofibrate, fluvastatin, lovastatin, pravastatin, simvastatin, rosuvastatin, ezetimibe, pitavastatin |
|  |  |
| Outcome |  |
| Anemia due to bleeding | D62 |
| Intracranial hemorrhage | I60-62 |
| Gastrointestinal bleeding | I85, K22.1, K22.8, K25.0, K25.4, K25.6, K26.0, K26.2,K26.4, K26.6, K27.0, K27.2, K27.4, K27.6, K28.0, K28.2, K28.4, K28.6, K29.0, K31.8, K55.2, K57.0, K57.1, K57.2, K57.3, K57.4, K57.8, K57.9, K62.5, K66.1, K92.0, K92.1, K92.2 |

eTable 2. Cancer types for each medication usage group

|  | antiplatelet | anticoagulant | combination |
| --- | --- | --- | --- |
| C00 |  |  |  |
| C01 |  |  |  |
| C02 | 4 |  |  |
| C03 | 1 | 1 |  |
| C04 |  | 1 |  |
| C05 | 2 |  |  |
| C06 | 2 |  |  |
| C07 | 2 |  |  |
| C08 | 1 |  | 1 |
| C09 | 4 |  |  |
| C10 | 1 |  |  |
| C11 | 2 |  |  |
| C12 | 1 |  | 1 |
| C13 | 3 |  |  |
| C14 | 1 |  |  |
| C15 | 17 | 3 | 3 |
| C16 | 236 | 22 | 18 |
| C17 | 4 | 30 | 1 |
| C18 | 152 |  | 22 |
| C19 | 10 | 2 | 2 |
| C20 | 104 | 13 | 11 |
| C21 | 10 |  | 1 |
| C22 | 142 | 19 | 18 |
| C23 | 46 | 3 | 5 |
| C24 | 42 | 8 | 7 |
| C25 | 76 | 14 | 11 |
| C26 | 2 | 1 |  |
| C27 |  |  |  |
| C28 |  |  |  |
| C29 |  |  |  |
| C30 | 1 | 1 |  |
| C31 | 2 |  |  |
| C32 | 11 | 1 | 1 |
| C33 |  |  |  |
| C34 | 323 | 41 | 36 |
| C35 |  |  |  |
| C36 |  |  |  |
| C37 |  |  |  |
| C38 | 1 | 1 |  |
| C39 | 1 |  |  |
| C40 |  |  |  |
| C41 | 2 | 1 |  |
| C42 |  |  |  |
| C43 | 1 | 3 |  |
| C44 | 21 |  |  |
| C45 | 1 |  |  |
| C46 |  |  |  |
| C47 | 1 |  |  |
| C48 | 3 | 1 |  |
| C49 | 3 |  |  |
| C50 | 95 | 15 | 13 |
| C51 | 2 | 1 |  |
| C52 | 2 |  |  |
| C53 | 52 | 8 | 3 |
| C54 | 5 | 1 | 1 |
| C55 | 3 |  |  |
| C56 | 16 | 4 | 2 |
| C57 | 2 | 1 |  |
| C58 |  |  |  |
| C59 |  |  |  |
| C60 | 1 |  |  |
| C61 | 153 | 23 | 14 |
| C62 |  |  |  |
| C63 |  |  |  |
| C64 | 17 | 1 |  |
| C65 | 4 | 2 |  |
| C66 | 8 |  | 1 |
| C67 | 74 | 8 | 10 |
| C68 | 2 |  |  |
| C69 |  |  |  |
| C70 |  |  |  |
| C71 | 7 |  | 1 |
| C72 |  |  |  |
| C73 | 33 | 4 | 10 |
| C74 |  |  |  |
| C75 |  |  |  |
| C76 | 6 |  |  |
| C77 | 7 | 1 |  |
| C78 | 14 | 3 | 6 |
| C79 | 16 | 1 |  |
| C80 | 6 | 1 |  |
| C81 | 1 |  |  |
| C82 | 4 |  |  |
| C83 | 11 | 2 | 2 |
| C84 | 3 |  |  |
| C85 | 8 | 2 | 1 |
| C86 |  |  |  |
| C87 |  |  |  |
| C88 | 2 |  |  |
| C89 |  |  |  |
| C90 | 7 | 1 | 1 |
| C91 | 6 | 1 |  |
| C92 | 17 | 5 | 1 |
| C93 | 1 |  |  |
| C94 | 1 | 1 |  |
| C95 | 6 |  |  |
| C96 | 1 |  |  |
| C97 |  |  |  |
| C98 |  |  |  |
| C99 |  |  |  |
| SUM | 1829 | 252 | 204 |

eTable 3. Bleeding events per each cancer types

|  | Major bleeding | ICH | GI bleeding | Anemia dute to acute bleeding |
| --- | --- | --- | --- | --- |
| C00 |  |  |  |  |
| C01 |  |  |  |  |
| C02 | 1 |  | 1 |  |
| C03 |  |  |  |  |
| C04 |  |  |  |  |
| C05 |  |  |  |  |
| C06 | 1 | 1 |  |  |
| C07 |  |  |  |  |
| C08 | 1 | 1 |  |  |
| C09 | 1 | 1 |  |  |
| C10 |  |  |  |  |
| C11 |  |  |  |  |
| C12 | 1 |  | 1 |  |
| C13 | 2 | 1 | 1 |  |
| C14 | 1 | 1 | 1 |  |
| C15 | 6 | 1 | 5 |  |
| C16 | 108 | 19 | 88 | 9 |
| C17 | 2 |  | 1 | 1 |
| C18 | 69 | 15 | 55 | 4 |
| C19 | 5 |  | 5 |  |
| C20 | 37 | 6 | 31 | 4 |
| C21 | 6 | 1 | 6 |  |
| C22 | 63 | 12 | 52 | 7 |
| C23 | 18 | 2 | 16 | 3 |
| C24 | 22 | 3 | 19 | 3 |
| C25 | 33 | 4 | 30 | 3 |
| C26 | 1 |  | 1 |  |
| C27 |  |  |  |  |
| C28 |  |  |  |  |
| C29 |  |  |  |  |
| C30 |  |  |  |  |
| C31 | 1 |  | 1 |  |
| C32 | 2 |  | 2 |  |
| C33 |  |  |  |  |
| C34 | 125 | 12 | 114 | 11 |
| C35 |  |  |  |  |
| C36 |  |  |  |  |
| C37 |  |  |  |  |
| C38 |  |  |  |  |
| C39 |  |  |  |  |
| C40 |  |  |  |  |
| C41 | 2 | 1 | 2 |  |
| C42 |  |  |  |  |
| C43 | 1 |  | 1 |  |
| C44 | 12 |  | 12 |  |
| C45 |  |  |  |  |
| C46 |  |  |  |  |
| C47 |  |  |  |  |
| C48 | 1 |  | 1 |  |
| C49 |  |  |  |  |
| C50 | 38 | 4 | 34 | 5 |
| C51 | 2 | 1 | 2 | 1 |
| C52 |  |  |  |  |
| C53 | 23 | 3 | 22 |  |
| C54 | 3 |  | 3 | 1 |
| C55 | 1 |  | 1 |  |
| C56 | 9 | 2 | 6 | 1 |
| C57 | 1 |  | 1 | 1 |
| C58 |  |  |  |  |
| C59 |  |  |  |  |
| C60 | 1 |  | 1 |  |
| C61 | 66 | 15 | 56 | 2 |
| C62 |  |  |  |  |
| C63 |  |  |  |  |
| C64 | 5 |  | 5 |  |
| C65 | 2 |  | 2 |  |
| C66 | 2 |  | 2 | 1 |
| C67 | 26 | 2 | 25 | 2 |
| C68 |  |  |  |  |
| C69 |  |  |  |  |
| C70 |  |  |  |  |
| C71 | 4 | 1 | 4 |  |
| C72 |  |  |  |  |
| C73 | 21 | 6 | 17 | 2 |
| C74 |  |  |  |  |
| C75 |  |  |  |  |
| C76 | 2 |  | 1 | 1 |
| C77 | 3 | 1 | 2 |  |
| C78 | 8 | 1 | 8 |  |
| C79 | 5 |  | 5 |  |
| C80 | 4 |  | 3 | 1 |
| C81 | 1 |  | 1 |  |
| C82 | 2 | 1 | 1 | 2 |
| C83 | 6 |  | 6 |  |
| C84 |  |  |  |  |
| C85 | 2 |  | 2 | 1 |
| C86 |  |  |  |  |
| C87 |  |  |  |  |
| C88 | 1 |  | 1 |  |
| C89 |  |  |  |  |
| C90 | 3 |  | 3 | 1 |
| C91 | 5 |  | 5 | 1 |
| C92 |  |  |  |  |
| C93 | 3 |  | 3 |  |
| C94 | 2 | 1 | 2 |  |
| C95 | 1 |  | 1 |  |
| C96 |  |  |  |  |
| C97 |  |  |  |  |
| C98 |  |  |  |  |
| C99 |  |  |  |  |
| SUM |  |  |  |  |

eTable 4. Subgroup analyses with demographic features, comorbidities, and cancer types

| **Age** | Young age: 19-54 years | N (total = 214) | Antiplatelet | Anticoagulation | Combination | Discontinuation | P for interaction |
| --- | --- | --- | --- | --- | --- | --- | --- |
|  | Major bleeding | 87 | 1 | 3.036 (2.285 - 4.033) | 6.827 (3.350 - 13.911) | 0.894 (0.743 - 1.076) | <0.0001 |
|  | Death | 103 | 1 | 1.613 (1.272 - 2.047) | 1.821 (0.970 - 3.418) | 1.185 (1.040 - 1.351) | 0.0005 |
|  |  |  |  |  |  |  |  |
|  | Middle age: 55-74 years | N (total = 1,341) | Antiplatelet | Anticoagulation | Combination | Discontinuation |  |
|  | Major bleeding | 444 | 1 | 0.967 (0.813 - 1.150) | 0.956 (0.599 - 1.527) | 0.695 (0.644 - 0.751) |  |
|  | Death | 837 | 1 | 1.069 (0.974 - 1.174) | 1.831 (1.495 - 2.243) | 1.180 (1.129 - 1.233) |  |
|  |  |  |  |  |  |  |  |
|  | Old age: ≥75 years | N (total = 730) | Antiplatelet | Anticoagulation | Combination | Discontinuation |  |
|  | Major bleeding | 243 | 1 | 1.577 (1.312 - 1.896) | 2.013 (1.224 - 3.310) | 0.665 (0.598 - 0.740) |  |
|  | Death | 591 | 1 | 0.904 (0.806 - 1.013) | 1.622 (1.255 - 2.097) | 1.207 (1.142 - 1.275) |  |
|  |  |  |  |  |  |  |  |
| **Sex** | Male | N (total = 1,581) | Antiplatelet | Anticoagulation | Combination | Discontinuation | P for interaction |
|  | Major bleeding | 549 | 1 | 1.347 (1.166 - 1.556) | 1.267 (0.886 - 1.811) | 0.720 (0.672 - 0.773) | 0.0029 |
|  | Death | 1,097 | 1 | 0.936 (0.858 - 1.022) | 1.601 (1.342 - 1.909) | 1.227 (1.181 - 1.275) | <0.0001 |
|  |  |  |  |  |  |  |  |
|  | Female | N (total = 704) | Antiplatelet | Anticoagulation | Combination | Discontinuation |  |
|  | Major bleeding | 225 | 1 | 1.369 (1.129 - 1.661) | 4.243 (2.333 - 7.717) | 0.668 (0.597 - 0.747) |  |
|  | Death | 434 | 1 | 1.229 (1.093 - 1.382) | 2.580 (1.876 - 3.550) | 1.084 (1.013 - 1.160) |  |
|  |  |  |  |  |  |  |  |
| **Hypertension** | HTN (no) | N (total = 722) | Antiplatelet | Anticoagulation | Combination | Discontinuation | P for interaction |
|  | Major bleeding | 230 | 1 | 1.227 (0.982 - 1.533) | 1.587 (0.847 - 2.972) | 0.641 (0.574 - 0.714) | 0.2129 |
|  | Death | 456 | 1 | 1.408 (1.249 - 1.587) | 1.883 (1.403 - 2.527) | 1.119 (1.051 - 1.191) | <0.0001 |
|  |  |  |  |  |  |  |  |
|  | HTN (yes) | N (total = 1,563) | Antiplatelet | Anticoagulation | Combination | Discontinuation |  |
|  | Major bleeding | 544 | 1 | 1.416 (1.234 - 1.624) | 1.532 (1.077 - 2.179) | 0.731 (0.681 - 0.785) |  |
|  | Death | 1,075 | 1 | 0.926 (0.85 - 1.009) | 1.703 (1.421 - 2.041) | 1.212 (1.165 - 1.261) |  |
|  |  |  |  |  |  |  |  |
| **Diabetes mellitus** | DM (no) | N (total = 1,652) | Antiplatelet | Anticoagulation | Combination | Discontinuation | P for interaction |
|  | Major bleeding | 570 | 1 | 1.179 (1.03 - 1.35) | 1.152 (0.781 - 1.699) | 0.681 (0.636 - 0.729) | <0.0001 |
|  | Death | 1,077 | 1 | 0.98 (0.901 - 1.064) | 1.691 (1.408 - 2.032) | 1.131 (1.085 - 1.178) | 0.0004 |
|  |  |  |  |  |  |  |  |
|  | DM (yes) | N (total = 633) | Antiplatelet | Anticoagulation | Combination | Discontinuation |  |
|  | Major bleeding | 204 | 1 | 2.332 (1.864 - 2.918) | 3.278 (1.981 - 5.426) | 0.778 (0.691 - 0.876) |  |
|  | Death | 454 | 1 | 1.223 (1.075 - 1.391) | 1.901 (1.429 - 2.530) | 1.295 (1.223 - 1.371) |  |
|  |  |  |  |  |  |  |  |
| **Dyslipidemia** | Dyslipidemia (no) | N (total = 1,684) | Antiplatelet | Anticoagulation | Combination | Discontinuation | P for interaction |
|  | Major bleeding | 565 | 1 | 1.512 (1.329 - 1.720) | 1.686 (1.216 - 2.340) | 0.721 (0.673 - 0.773) | 0.0016 |
|  | Death | 1,126 | 1 | 1.015 (0.936 - 1.100) | 1.804 (1.524 - 2.135) | 1.205 (1.159 - 1.254) | 0.0287 |
|  |  |  |  |  |  |  |  |
|  | Dyslipidemia (yes) | N (total = 601) | Antiplatelet | Anticoagulation | Combination | Discontinuation |  |
|  | Major bleeding | 209 | 1 | 0.833 (0.622 - 1.117) | 0.950 (0.392 - 2.304) | 0.656 (0.585 - 0.736) |  |
|  | Death | 405 | 1 | 1.184 (1.029 - 1.362) | 1.574 (1.073 - 2.310) | 1.133 (1.063 - 1.207) |  |
|  |  |  |  |  |  |  |  |
| **Coronary artery disease** | CAD (no) | N (total = 1,586) | Antiplatelet | Anticoagulation | Combination | Discontinuation | P for interaction |
|  | Major bleeding | 532 | 1 | 1.517 (1.319 - 1.744) | 1.325 (0.891 - 1.970) | 0.706 (0.658 - 0.758) | 0.0247 |
|  | Death | 1,049 | 1 | 1.088 (0.996 - 1.189) | 1.498 (1.220 - 1.840) | 1.178 (1.131 - 1.227) | 0.0231 |
|  |  |  |  |  |  |  |  |
|  | CAD (yes) | N (total = 699) | Antiplatelet | Anticoagulation | Combination | Discontinuation |  |
|  | Major bleeding | 242 | 1 | 1.099 (0.897 - 1.347) | 1.975 (1.214 - 3.213) | 0.696 (0.624 - 0.775) |  |
|  | Death | 482 | 1 | 0.988 (0.883 - 1.106) | 2.253 (1.783 - 2.847) | 1.199 (1.131 - 1.272) |  |
|  |  |  |  |  |  |  |  |
| **Atrial fibrillation** | AF (no) | N (total = 2,064) | Antiplatelet | Anticoagulation | Combination | Discontinuation | P for interaction |
|  | Major bleeding | 706 | 1 | 1.591 (1.383 - 1.830) | 1.889 (1.363 - 2.618) | 0.692 (0.650 - 0.736) | <0.0001 |
|  | Death | 1,375 | 1 | 1.446 (1.329 - 1.573) | 1.968 (1.632 - 2.373) | 1.152 (1.112 - 1.193) | <0.0001 |
|  |  |  |  |  |  |  |  |
|  | AF (yes) | N (total = 221) | Antiplatelet | Anticoagulation | Combination | Discontinuation |  |
|  | Major bleeding | 68 | 1 | 1.161 (0.888 - 1.519) | 0.658 (0.267 - 1.626) | 0.851 (0.662 - 1.094) |  |
|  | Death | 156 | 1 | 0.786 (0.682 - 0.906) | 1.509 (1.137 - 2.003) | 1.597 (1.408 - 1.811) |  |
|  |  |  |  |  |  |  |  |
| **Valvular heart disease** | VHD (no) | N (total = 2,257) | Antiplatelet | Anticoagulation | Combination | Discontinuation | P for interaction |
|  | Major bleeding | 765 | 1 | 1.386 (1.228 - 1.565) | 1.531 (1.121 - 2.089) | 0.699 (0.658 - 0.743) | 0.0652 |
|  | Death | 1,511 | 1 | 1.081 (1.004 - 1.164) | 1.705 (1.454 - 2.000) | 1.174 (1.135 - 1.214) | <0.0001 |
|  |  |  |  |  |  |  |  |
|  | VHD (yes) | N (total = 28) | Antiplatelet | Anticoagulation | Combination | Discontinuation |  |
|  | Major bleeding | 9 | 1 | 1.325 (0.666 - 2.636) | 2.135 (0.279 - 16.353) | 1.293 (0.687 - 2.434) |  |
|  | Death | 20 | 1 | 0.934 (0.678 - 1.287) | 4.078 (2.104 - 7.902) | 2.216 (1.676 - 2.929) |  |
|  |  |  |  |  |  |  |  |
| **Heart failure** | HF (no) | N (total = 2,091) | Antiplatelet | Anticoagulation | Combination | Discontinuation | P for interaction |
|  | Major bleeding | 712 | 1 | 1.351 (1.187 - 1.537) | 1.409 (0.963 - 2.062) | 0.713 (0.670 - 0.759) | 0.3559 |
|  | Death | 1,387 | 1 | 1.057 (0.978 - 1.143) | 1.839 (1.547 - 2.187) | 1.189 (1.148 - 1.231) | 0.5697 |
|  |  |  |  |  |  |  |  |
|  | HF (yes) | N (total = 194) | Antiplatelet | Anticoagulation | Combination | Discontinuation |  |
|  | Major bleeding | 62 | 1 | 1.319 (0.997 - 1.745) | 1.719 (1.008 - 2.932) | 0.609 (0.496 - 0.747) |  |
|  | Death | 144 | 1 | 0.973 (0.820 - 1.154) | 1.462 (1.035 - 2.064) | 1.132 (1.007 - 1.273) |  |
|  |  |  |  |  |  |  |  |
| **Gastro-intestinal ulcer** | GI ulcer (no) | N (total = 759) | Antiplatelet | Anticoagulation | Combination | Discontinuation | P for interaction |
|  | Major bleeding | 260 | 1 | 1.484 (1.235 - 1.782) | 0.580 (0.260 - 1.297) | 0.694 (0.626 - 0.768) | 0.0142 |
|  | Death | 513 | 1 | 1.075 (0.962 - 1.201) | 0.951 (0.698 - 1.297) | 1.135 (1.072 - 1.202) | <0.0001 |
|  |  |  |  |  |  |  |  |
|  | GI ulcer (yes) | N (total = 1,526) | Antiplatelet | Anticoagulation | Combination | Discontinuation |  |
|  | Major bleeding | 514 | 1 | 1.295 (1.117 - 1.501) | 2.125 (1.522 - 2.967) | 0.709 (0.659 - 0.763) |  |
|  | Death | 1,018 | 1 | 1.025 (0.938 - 1.120) | 2.402 (2.011 - 2.869) | 1.210 (1.162 - 1.261) |  |
|  |  |  |  |  |  |  |  |
| **Deep vein thrombosis** | DVT (no) | N (total = 2,254) | Antiplatelet | Anticoagulation | Combination | Discontinuation | P for interaction |
|  | Major bleeding | 761 | 1 | 1.365 (1.209 - 1.541) | 1.648 (1.212 - 2.241) | 0.714 (0.672 - 0.759) | 0.0148 |
|  | Death | 1,506 | 1 | 1.039 (0.966 - 1.117) | 1.757 (1.502 - 2.055) | 1.192 (1.152 - 1.233) | 0.0031 |
|  |  |  |  |  |  |  |  |
|  | DVT (yes) | N (total = 31) | Antiplatelet | Anticoagulation | Combination | Discontinuation |  |
|  | Major bleeding | 5 | 1 | 1.110 (0.607 - 2.028) | NA | 0.404 (0.287 - 0.569) |  |
|  | Death | 13 | 1 | 1.356 (0.876 - 2.098) | 1.725 (0.634 - 4.689) | 0.807 (0.619 - 1.052) |  |
|  |  |  |  |  |  |  |  |
| **Pulmonary thrombo-embolism** | PTE (no) | N (total = 2,269) | Antiplatelet | Anticoagulation | Combination | Discontinuation | P for interaction |
|  | Major bleeding | 230 | 1 | 1.371 (1.216 - 1.546) | 1.540 (1.132 - 2.095) | 0.705 (0.664 - 0.749) | 0.6036 |
|  | Death | 446 | 1 | 1.053 (0.979 - 1.133) | 1.751 (1.499 - 2.045) | 1.185 (1.146 - 1.226) | 0.0067 |
|  |  |  |  |  |  |  |  |
|  | PTE (yes) | N (total = 16) | Antiplatelet | Anticoagulation | Combination | Discontinuation |  |
|  | Major bleeding | 544 | 1 | 0.584 (0.161 - 2.123) | NA | 0.494 (0.224 - 1.090) |  |
|  | Death | 1,085 | 1 | 0.621 (0.308 - 1.248) | 38.980 (5.278 - 287.850) | 1.070 (0.706 - 1.621) |  |
|  |  |  |  |  |  |  |  |
| **7 major cancer type** | No | N (total = 670) | Antiplatelet | Anticoagulation | Combination | Discontinuation | P for interaction |
|  | Major bleeding | 230 | 1 | 1.709 (1.412 - 2.067) | 2.578 (1.678 - 3.961) | 0.795 (0.713 - 0.886) | 0.0006 |
|  | Death | 446 | 1 | 0.962 (0.847 - 1.093) | 2.864 (2.237 - 3.666) | 1.261 (1.182 - 1.346) | <0.0001 |
|  |  |  |  |  |  |  |  |
|  | Yes | N (total = 1,615) | Antiplatelet | Anticoagulation | Combination | Discontinuation |  |
|  | Major bleeding | 544 | 1 | 1.216 (1.051 - 1.406) | 1.082 (0.695 - 1.685) | 0.671 (0.625 - 0.720) |  |
|  | Death | 1,085 | 1 | 1.078 (0.991 - 1.172) | 1.401 (1.150 - 1.707) | 1.164 (1.120 - 1.211) |  |
|  |  |  |  |  |  |  |  |
| **Stomach cancer** | No | N (total = 2,009) | Antiplatelet | Anticoagulation | Combination | Discontinuation | P for interaction |
|  | Major bleeding | 666 | 1 | 1.294 (1.139 - 1.470) | 1.61 (1.175 - 2.206) | 0.703 (0.659 - 0.749) | 0.0361 |
|  | Death | 1,348 | 1 | 1.023 (0.948 - 1.103) | 1.852 (1.574 - 2.180) | 1.176 (1.135 - 1.219) | 0.0560 |
|  |  |  |  |  |  |  |  |
|  | Yes | N (total = 276) | Antiplatelet | Anticoagulation | Combination | Discontinuation |  |
|  | Major bleeding | 108 | 1 | 1.996 (1.477 - 2.698) | 0.811 (0.201 - 3.269) | 0.713 (0.604 - 0.841) |  |
|  | Death | 183 | 1 | 1.278 (1.033 - 1.582) | 1.208 (0.746 - 1.958) | 1.245 (1.132 - 1.368) |  |
|  |  |  |  |  |  |  |  |
| **Colorectal cancer** | No | N (total = 1,939) | Antiplatelet | Anticoagulation | Combination | Discontinuation | P for interaction |
|  | Major bleeding | 663 | 1 | 1.469 (1.293 - 1.669) | 1.637 (1.138 - 2.355) | 0.715 (0.669 - 0.765) | 0.0227 |
|  | Death | 1,297 | 1 | 1.061 (0.983 - 1.146) | 1.934 (1.625 - 2.301) | 1.199 (1.156 - 1.244) | 0.0536 |
|  |  |  |  |  |  |  |  |
|  | Yes | N (total = 346) | Antiplatelet | Anticoagulation | Combination | Discontinuation |  |
|  | Major bleeding | 111 | 1 | 0.908 (0.672 - 1.227) | 1.129 (0.635 - 2.007) | 0.660 (0.578 - 0.754) |  |
|  | Death | 234 | 1 | 0.969 (0.814 - 1.153) | 1.228 (0.876 - 1.721) | 1.109 (1.022 - 1.204) |  |
|  |  |  |  |  |  |  |  |
| **Liver cancer** | No | N (total = 2,106) | Antiplatelet | Anticoagulation | Combination | Discontinuation | P for interaction |
|  | Major bleeding | 711 | 1 | 1.319 (1.166 - 1.492) | 1.496 (1.100 - 2.035) | 0.700 (0.658 - 0.745) | 0.1489 |
|  | Death | 1,411 | 1 | 1.045 (0.970 - 1.126) | 1.770 (1.513 - 2.070) | 1.182 (1.141 - 1.223) | 0.8352 |
|  |  |  |  |  |  |  |  |
|  | Yes | N (total = 179) | Antiplatelet | Anticoagulation | Combination | Discontinuation |  |
|  | Major bleeding | 63 | 1 | 2.154 (1.436 - 3.231) | NA | 0.784 (0.608 - 1.012) |  |
|  | Death | 120 | 1 | 1.070 (0.857 - 1.337) | 1.233 (0.460 - 3.308) | 1.224 (1.083 - 1.383) |  |
|  |  |  |  |  |  |  |  |
| **Pancreas cancer** | No | N (total = 2,184) | Antiplatelet | Anticoagulation | Combination | Discontinuation | P for interaction |
|  | Major bleeding | 741 | 1 | 1.400 (1.240 - 1.579) | 1.661 (1.221 - 2.259) | 0.710 (0.667 - 0.755) | 0.0721 |
|  | Death | 1,463 | 1 | 1.015 (0.942 - 1.093) | 1.769 (1.508 - 2.076) | 1.188 (1.148 - 1.230) | 0.0001 |
|  |  |  |  |  |  |  |  |
|  | Yes | N (total = 101) | Antiplatelet | Anticoagulation | Combination | Discontinuation |  |
|  | Major bleeding | 33 | 1 | 0.444 (0.180 - 1.095) | NA | 0.579 (0.433 - 0.773) |  |
|  | Death | 68 | 1 | 1.747 (1.338 - 2.279) | 1.575 (0.860 - 2.884) | 1.113 (0.951 - 1.303) |  |
|  |  |  |  |  |  |  |  |
| **Lung cancer** | No | N (total = 1,885) | Antiplatelet | Anticoagulation | Combination | Discontinuation | P for interaction |
|  | Major bleeding | 649 | 1 | 1.471 (1.296 - 1.669) | 1.665 (1.209 - 2.293) | 0.716 (0.670 - 0.764) | 0.0126 |
|  | Death | 1,271 | 1 | 1.037 (0.959 - 1.121) | 1.707 (1.443 - 2.020) | 1.184 (1.141 - 1.228) | 0.7648 |
|  |  |  |  |  |  |  |  |
|  | Yes | N (total = 400) | Antiplatelet | Anticoagulation | Combination | Discontinuation |  |
|  | Major bleeding | 125 | 1 | 0.865 (0.625 - 1.196) | 0.760 (0.244 - 2.369) | 0.648 (0.561 - 0.750) |  |
|  | Death | 260 | 1 | 1.088 (0.923 - 1.283) | 2.104 (1.415 - 3.128) | 1.190 (1.100 - 1.287) |  |
|  |  |  |  |  |  |  |  |
| **Breast cancer** | No | N (total = 2,162) | Antiplatelet | Anticoagulation | Combination | Discontinuation | P for interaction |
|  | Major bleeding | 736 | 1 | 1.354 (1.199 - 1.530) | 1.415 (1.029 - 1.947) | 0.707 (0.665 - 0.752) | 0.0017 |
|  | Death | 1,438 | 1 | 1.068 (0.992 - 1.150) | 1.760 (1.505 - 2.058) | 1.193 (1.152 - 1.235) | 0.0087 |
|  |  |  |  |  |  |  |  |
|  | Yes | N (total = 123) | Antiplatelet | Anticoagulation | Combination | Discontinuation |  |
|  | Major bleeding | 38 | 1 | 1.562 (0.942 - 2.592) | 14.337 (4.492 - 45.764) | 0.679 (0.501 - 0.922) |  |
|  | Death | 93 | 1 | 0.736 (0.567 - 0.956) | 3.549 (1.325 - 9.507) | 1.034 (0.905 - 1.181) |  |
|  |  |  |  |  |  |  |  |
| **Prostate cancer** | No | N (total = 2,095) | Antiplatelet | Anticoagulation | Combination | Discontinuation | P for interaction |
|  | Major bleeding | 708 | 1 | 1.345 (1.187 - 1.525) | 1.624 (1.194 - 2.209) | 0.705 (0.662 - 0.751) | 0.9133 |
|  | Death | 1,404 | 1 | 1.053 (0.977 - 1.136) | 1.804 (1.541 - 2.112) | 1.196 (1.154 - 1.238) | 0.2533 |
|  |  |  |  |  |  |  |  |
|  | Yes | N (total = 190) | Antiplatelet | Anticoagulation | Combination | Discontinuation |  |
|  | Major bleeding | 66 | 1 | 1.507 (1.069 - 2.126) | NA | 0.695 (0.577 - 0.838) |  |
|  | Death | 127 | 1 | 0.986 (0.787 - 1.235) | 1.134 (0.506 - 2.540) | 1.078 (0.967 - 1.203) |  |

eTable 5. Comparison of major bleeding and death incidence between drug-exposed types without atrial fibrillation diagnosis

| Unadjusted | N (total = 2,064) | Antiplatelet | Anticoagulation | Combination | Discontinuation |
| --- | --- | --- | --- | --- | --- |
| ICH | 107 (5.2) | 1 | 1.885 (1.397 - 2.544) | 2.668 (1.387 - 5.209) | 0.646 (0.558 - 0.747) |
| GI bleeding | 615 (29.8) | 1 | 1.510 (1.302 - 1.752) | 2.335 (1.686 - 3.234) | 0.677 (0.634 - 0.722) |
| Major bleeding | 706 (34.2) | 1 | 1.582 (1.376 - 1.818) | 2.140 (1.546 - 2.968) | 0.696 (0.654 - 0.741) |
| Death | 1,375 (66.6) | 1 | 1.367 (1.257 - 1.486) | 1.931 (1.601 - 2.328) | 1.118 (1.080 - 1.158) |
|  |  |  |  |  |  |
| Model 1* | N (total = 2,064) | Antiplatelet | Anticoagulation | Combination | Discontinuation |
| ICH | 107 (5.2) | 1 | 1.724 (1.275 - 2.330) | 2.970 (1.532 - 5.760) | 0.652 (0.564 - 0.755) |
| GI bleeding | 615 (29.8) | 1 | 1.515 (1.305 - 1.758) | 2.317 (1.672 - 3.209) | 0.679 (0.637 - 0.725) |
| Major bleeding | 706 (34.2) | 1 | 1.571 (1.366 - 1.806) | 2.150 (1.553 - 2.978) | 0.699 (0.657 - 0.743) |
| Death | 1,375 (66.6) | 1 | 1.442 (1.327 - 1.568) | 1.776 (1.473 - 2.141) | 1.145 (1.105 - 1.185) |
|  |  |  |  |  |  |
| Model 2† | N (total = 2,064) | Antiplatelet | Anticoagulation | Combination | Discontinuation |
| ICH | 107 (5.2) | 1 | 1.182 (0.862 - 1.622) | 2.386 (1.227 - 4.640) | 0.603 (0.521 - 0.698) |
| GI bleeding | 615 (29.8) | 1 | 1.472 (1.267 - 1.711) | 2.136 (1.540 - 2.962) | 0.674 (0.632 - 0.719) |
| Major bleeding | 706 (34.2) | 1 | 1.520 (1.320 - 1.751) | 1.902 (1.372 - 2.638) | 0.690 (0.649 - 0.735) |
| Death | 1,375 (66.6) | 1 | 1.475 (1.356 - 1.605) | 1.839 (1.524 - 2.218) | 1.154 (1.114 - 1.195) |

* Age, sex adjusted

† Age, sex, hypertension, diabetes mellitus, dyslipidemia, atrial fibrillation, coronary artery disease, heart failure, valvular heart disease, deep venous thrombosis, pulmonary thromboembolism, and gastrointestinal ulcer adjusted
